# Hypothalamic structural integrity and temporal complexity of cortical information processing at rest in migraine without aura patients between attacks

**DOI:** 10.1038/s41598-021-98213-3

**Published:** 2021-09-21

**Authors:** Camillo Porcaro, Antonio Di Renzo, Emanuele Tinelli, Giorgio Di Lorenzo, Stefano Seri, Cherubino Di Lorenzo, Vincenzo Parisi, Francesca Caramia, Marco Fiorelli, Vittorio Di Piero, Francesco Pierelli, Gianluca Coppola

**Affiliations:** 1 Institute of Cognitive Sciences and Technologies (ISTC) - National Research Council (CNR), Rome, Italy; 2 Centre for Human Brain Health and School of Psychology, University of Birmingham, Birmingham, UK; 3 S. Anna Institute and Research in Advanced Neurorehabilitation (RAN), Crotone, Italy; 4 Department of Information Engineering - Università Politecnica delle Marche, Ancona, Italy; 5 IRCCS Fondazione Bietti, Rome, Italy; 6 Department of Human Neurosciences, Sapienza University of Rome, Rome, Italy; 7 Laboratory of Psychophysiology and Cognitive Neuroscience, Department of Systems Medicine, University of Rome Tor Vergata, Rome, Italy; 8 IRCCS - Fondazione Santa Lucia, Rome, Italy; 9 College of Health and Life Sciences, Aston Institute of Health and Neurodevelopment, Aston University, Birmingham, UK; 10 Department of Clinical Neurophysiology, Birmingham Women’s and Children’s NHS Foundation Trust, Birmingham, UK; 11 Department of Medico-Surgical Sciences and Biotechnologies, Sapienza University of Rome Polo Pontino, Corso della Repubblica 79, 04100 Latina, Italy; 12 IRCCS - Neuromed, Pozzilli, IS Italy

**Keywords:** Neuroscience, Cognitive neuroscience, Computational neuroscience, Sensory processing

## Abstract

The hypothalamus has been attributed an important role during the premonitory phase of a migraine attack. Less is known about the role played by the hypothalamus in the interictal period and its relationship with the putative neurocognitive networks previously identified in the pathophysiology of migraine. Our aim was to test whether the hypothalamic microstructure would be altered during the interictal period and whether this co-existed with aberrant connectivity at cortical level. We collected multimodal MRI data from 20 untreated patients with migraine without aura between attacks (MO) and 20 healthy controls (HC) and studied fractional anisotropy, mean (MD), radial (RD), and axial diffusivity of the hypothalamus ROI as a whole from diffusion tensor imaging (DTI). Moreover, we performed an exploratory analysis of the same DTI metrics separately for the anterior and posterior hypothalamic ROIs bilaterally. From resting-state functional MRI, we estimated the Higuchi’s fractal dimension (FD), an index of temporal complexity sensible to describe non-periodic patterns characterizing BOLD signature. Finally, we correlated neuroimaging findings with migraine clinical features. In comparison to HC, MO had significantly higher MD, AD, and RD values within the hypothalamus. These findings were confirmed also in the exploratory analysis on the sub-regions of the hypothalamus bilaterally, with the addition of lower FA values on the posterior ROIs. Patients showed higher FD values within the salience network (SN) and the cerebellum, and lower FD values within the primary visual (PV) network compared to HC. We found a positive correlation between cerebellar and SN FD values and severity of migraine. Our findings of hypothalamic abnormalities between migraine attacks may form part of the neuroanatomical substrate that predisposes the onset of the prodromal phase and, therefore, the initiation of an attack. The peculiar fractal dimensionality we found in PV, SN, and cerebellum may be interpreted as an expression of abnormal efficiency demand of brain networks devoted to the integration of sensory, emotional, and cognitive information related to the severity of migraine.

## Introduction

In recent years, studies of the pathophysiology of migraine have seen a renewed interest in the role played by the hypothalamus in the recurrence of migraine attacks. This brain structure seems to play a predominant role during the phase immediately preceding an attack when some patients experience premonitory symptoms^1–3^. During the attack, the hypothalamus is metabolically hyperactive^4^, poorly connected to the spinal trigeminal nucleus^2^, and more strongly coupled with the dorsal pons^5^. The hypothalamus also appears to be involved in the state of a never-ending migraine attack that is chronic migraine^6,7^.

Furthermore, the interictal phase is characterized by abnormal connectivity of cortical networks and this has been shown to correlate with clinical variables such as frequency of attacks and severity of migraine pain^8^. We recently reported that abnormal functional connectivity between some of these networks and the hypothalamus is peculiar to patients suffering from chronic migraine^7^. Moulton and colleagues^9^ using an ROI-to-ROI approach, found stronger functional connectivity between the hypothalamus and several brain regions involved in the regulation of autonomic functions in interictal migraine patients but little is known at whole-brain level.

Here, we sought whether there is abnormal integrity of the hypothalamic structures and of functional connectivity of cortical networks in a group of migraine patients without aura between attacks. Therefore, we measure the hypothalamic microstructure through diffusion tensor imaging (DTI), a useful sensitive method to detect white matter tracts in grey matter nuclei—as is the case for the hypothalamus^10^ and the independent cortical networking by acquiring resting-state functional MRI taking the advantages of the non-linearity of the Higuchi's fractal dimension (FD) analysis^11–14^. This non-linear approach is more suitable to describe the irregular and non-periodic patterns characterizing the BOLD signature of discrete cortical areas belonging to a resting-state network (RSN) recorded by neuroimaging^15,16^ as well as electrophysiological techniques^17,18^.

Considering that in previous studies the hypothalamus has been involved in the pre-ictal period of migraine and that the activity of cortical networks is dysfunctional even outside attacks, we hypothesized that the microstructure of the hypothalamus could be altered during the pain-free period, as a favoring anatomical substrate to the recurrence of migraine, and that would be independent from aberrant RSN connectivity and migraine clinical features.

## Results

Demographic characteristics of MO and HC and clinical features of MO are summarized in Table 1. No significant difference emerged between MO and HC in gender (${\chi }_{1}^{2}$ = 0.102, p = 0.749) and age (*t*_38_ = − 1.628, p = 0.112).

**Table 1:** Clinical and demographic of healthy controls (HC) and of patients with migraine without aura (MO).

	HC (N = 20)	MO (N = 20)
Women (number)	11	12
Age (years)	29 ± 4	32 ± 7
Attacks frequency/month (number)		3 ± 2.0
Disease duration (year)		14 ± 6.5
Severity of headache (0–10)		7.3 ± 0.9
Headache-related disability (number)		2 ± 0.4
Tablet intake/month (number)		3 ± 1.8
Days since the last migraine attack (number)		21 ± 17.5

Data are expressed as frequency and mean ± SD.

In patients with MO, we did not detect white matter lesions.

### Characterization of hypothalamic DTI

No multivariate outliers were present in the MANOVA model (highest Mahalanobis Distance value: 14.843). Multivariate test revealed a significant *GROUPs* effect (Wilks' *λ* = 0.736, F_4,35_ = 3.131, p = 0.027). Univariate ANOVA analyses showed that, compared to HC, patients with MO showed significantly higher MD, AD, and RD hypothalamic DTI metrics, with large effect sizes. FA DTI metric did not statistically differ between groups, even though the effect size was moderate to large. In Table 2, descriptive and univariate statistics of DTI metrics for hypothalamic ROI are reported.

**Table 2:** Descriptive and univariate statistics for the hypothalamus fractional anisotropy (FA), mean diffusivity (MD), axial diffusivity (AD), and radial diffusivity (RD) in HC and MO.

DTI metric	HC	MO	Statistics
FA	0.271 ± 0.050	0.241 ± 0.048	F_1,38_ = 3.650, p = 0.064 *d* = 0.62
MD	1.170E−03 ± 1.630E−04	1.300E−03 ± 1.740E−04	F_1,38_ = 5.829, p = 0.021 *d* = − 0.79
AD	1.450E−03 ± 1.530E−04	1.570E−03 ± 1.700E−04	F_1,38_ = 5.329, p = 0.027 *d* = − 0.76
RD	1.030E−03 ± 1.690E−04	1.170E−03 ± 1.780E−04	F_1,38_ = 7.001, p = 0.012 *d* = − 0.83

Regarding the DTI metrics in the four regions of interest of hypothalamus, in the rm-MANOVA model no multivariate outliers were present (highest Mahalanobis Distance value: 32.307). Multivariate test revealed a *PARTs* × *SIDEs* × *GROUPs* effect just above the level of significance (Wilks' *λ* = 0.779, F_4,35_ = 2.487, p = 0.061). Despite Wilks’ Lambda multivariate significance criterion not being reached, we proceeded to univariate analysis for exploratory purposes. The rm-ANOVA model of FA did not have a significant *PARTs* × *SIDEs* × *GROUPs* effect (F_1,38_ = 0.099, p = 0.755) whereas the rm-ANOVA models for MD, AD, and RD revealed significant *PARTs* × *SIDEs* × *GROUPs* effects (respectively: MD: F_1,38_ = 7.674, p = 0.009; AD: F_1,38_ = 8.670, p = 0.005; RD: F_1,38_ = 6.076, p = 0.018). Univariate ANOVA analyses revealed that FA in the anterior part of the left and right hypothalamus did not differ between HC and MO whereas FA in the posterior part of the left and right hypothalamus was significantly lower in patients with MO than HC, with large effect sizes (respectively: left posterior, *d* = 1.36; right posterior, *d* = 1.81). Consistently, MD, AD, and RD in the anterior and posterior part of the left and right hypothalamus were significantly higher in MO compared to HV, with large to huge effect sizes [*d*s ranging from 0.85 (AD in left anterior hypothalamus) to 2.89 (AD in left posterior hypothalamus)]. In Table 3, descriptive and univariate statistics of DTI metrics of the anterior and posterior part of the left and right hypothalamus in HC and MO are reported.

**Table 3:** Descriptive and univariate statistics for the fractional anisotropy (FA), mean diffusivity (MD), axial diffusivity (AD), and radial diffusivity (RD) of the anterior and posterior part of the left and right hypothalamus in HC and MO.

DTI metric	HC	MO	Statistics
**FA**			
Left anterior	0.172 ± 0.051	0.197 ± 0.120	F_1,38_ = 0.766, p = 0.387 *d* = − 0.28
Left posterior	0.273 ± 0.149	0.115 ± 0.079	F_1,38_ = 17.509, p = 0.0002 *d* = 1.36
Right anterior	0.178 ± 0.061	0.176 ± 0.099	F_1,38_ = 0.003, p = 0.960 *d* = 0.02
Right posterior	0.316 ± 0.124	0.124 ± 0.091	F_1,38_ = 31.175, p < 0.0001 *d* = 1.81
**MD**			
Left anterior	1.509E−03 ± 3.580E−04	2.077E−03 ± 6.780E−04	F_1,38_ = 10.984, p = 0.002 *d* = − 1.07
Left posterior	1.576E−03 ± 4.830E−04	2.914E−03 ± 4.980E−04	F_1,38_ = 74.384, p < 0.0001 *d* = − 2.80
Right anterior	1.360E−03 ± 2.510E−04	2.056E−03 ± 7.850E−−04	F_1,38_ = 14.278, p = 0.001 *d* = − 1.23
Right posterior	1.744E−03 ± 5.740E−04	2.859E−03 ± 5.320E−04	F_1,38_ = 40.587, p < 0.0001 *d* = − 2.07
**AD**			
Left anterior	1.689E−03 ± 4.270E−04	2.224E−03 ± 8.060E−04	F_1,38_ = 6.888, p = 0.012 *d* = − 0.85
Left posterior	1.828E−03 ± 4.780E−04	3.202E−03 ± 4.960E−04	F_1,38_ = 79.542, p < 0.0001 *d* = − 2.89
Right anterior	1.514E−03 ± 2.690E−04	2.252E−03 ± 7.650E−04	F_1,38_ = 16.583, p = 0.0002 *d* = − 1.32
Right posterior	2.072E−03 ± 5.580E−04	3.180E−03 ± 4.180E−04	F_1,38_ = 50.473, p < 0.0001 *d* = − 2.31
**RD**			
Left anterior	1.431E−03 ± 3.070E−04	1.975E−03 ± 7.200E−04	F_1,38_ = 9.655, p = 0.004 *d* = − 1.01
Left posterior	1.471E−03 ± 4.980E−04	2.776E−03 ± 5.570E−04	F_1,38_ = 61.077, p < 0.0001 *d* = − 2.53
Right anterior	1.282E−03 ± 2.420E−04	1.906E−03 ± 7.650E−04	F_1,38_ = 12.081, p = 0.001 *d* = − 1.13
Right posterior	1.600E−03 ± 5.770E−04	2.693E−03 ± 5.560E−04	F_1,38_ = 37.177, p < 0.0001 *d* = − 1.98

### fMRI resting-state networks

**Figure 1:**
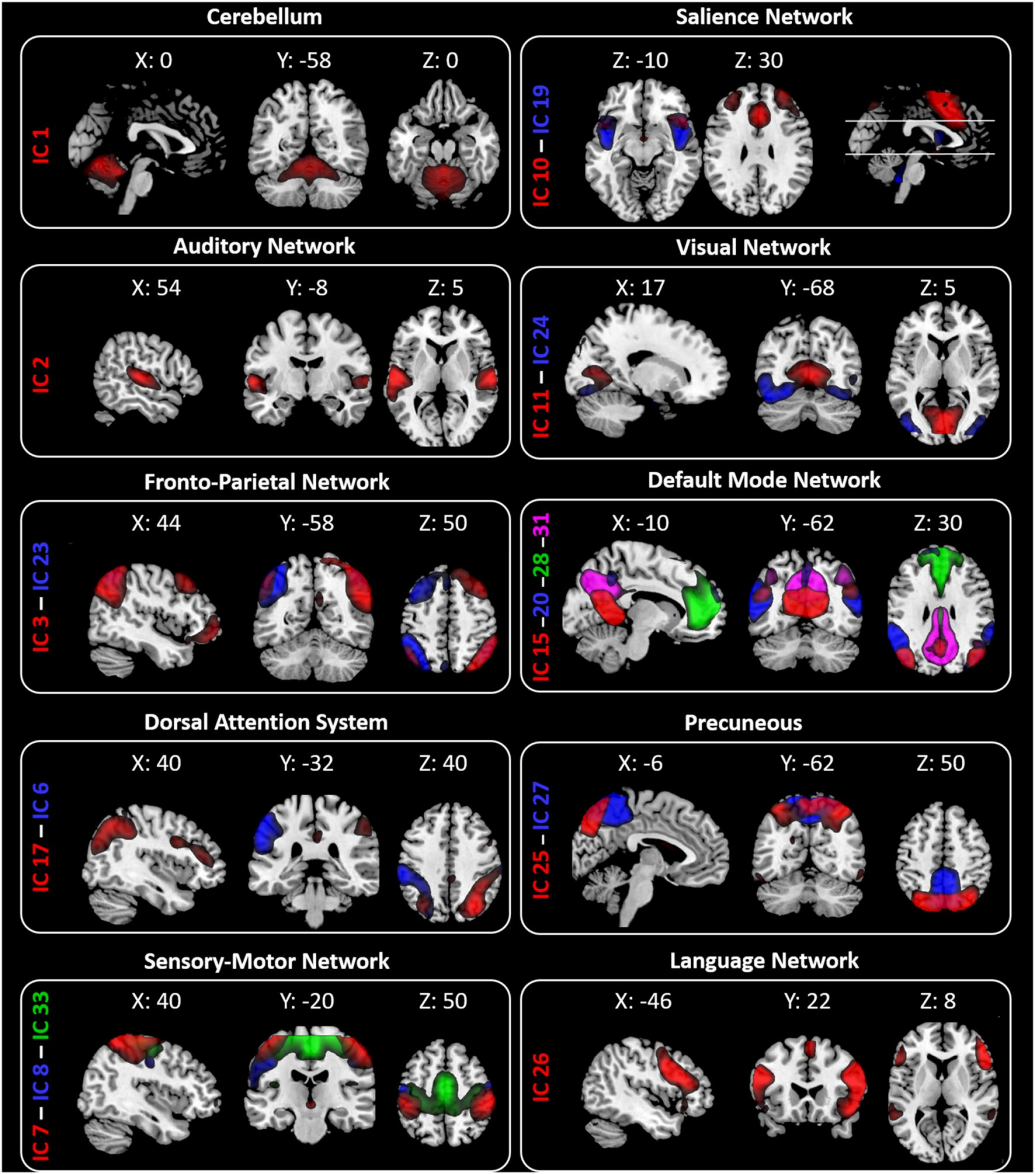
Resting State Networks (RSNs) identified by GIFT. Twenty spatial maps divided into ten functional networks were found: Cerebellum (Cb—IC1); Auditory (AN—IC2); Fronto-Parietal (FPN: IC3—rFPN and IC23—lFPN); Dorsal Attention System (DAS: IC6—lDAS and IC17—rDAS); Sensory Motor (SMN—IC7, IC8, IC33); Salience (SN—IC 10 and IC19); Visual (VN: IC11—Primary Visual and IC24—Lateral Visual); Default Mode (DMN—IC15, IC20, IC28 and IC31); Precuneous (IC25 and IC27) and Language (LN—IC 26) networks based on their anatomical view. Montreal Neurological Institute (MNI) coordinates are shown as well.

The twenty ICs were grouped into the following ten large-scale networks based on their spatial patterns (Fig. 1): Cerebellum (IC1—Cb); Auditory Network (IC2—AN); Fronto-Parietal Network (IC3—right FPN and IC23—Left FPN); Dorsal Attention System (IC6—left DAS and IC17—right DAS); Sensory Motor Network (IC7, IC8, IC33); Salience Network (IC 10—anterior part of SN (aSN) and IC19); Visual Network (IC11—Primary visual (PV) and IC 24—lateral visual (LVN)); Default Mode Network (DMN—IC15, IC20, IC28, and IC31); Precuneous (IC25 and IC27); Language Network (LN—IC 26).

### Characterization of the BOLD RSNs by Higuchi’s fractal dimension

**Figure 2:**
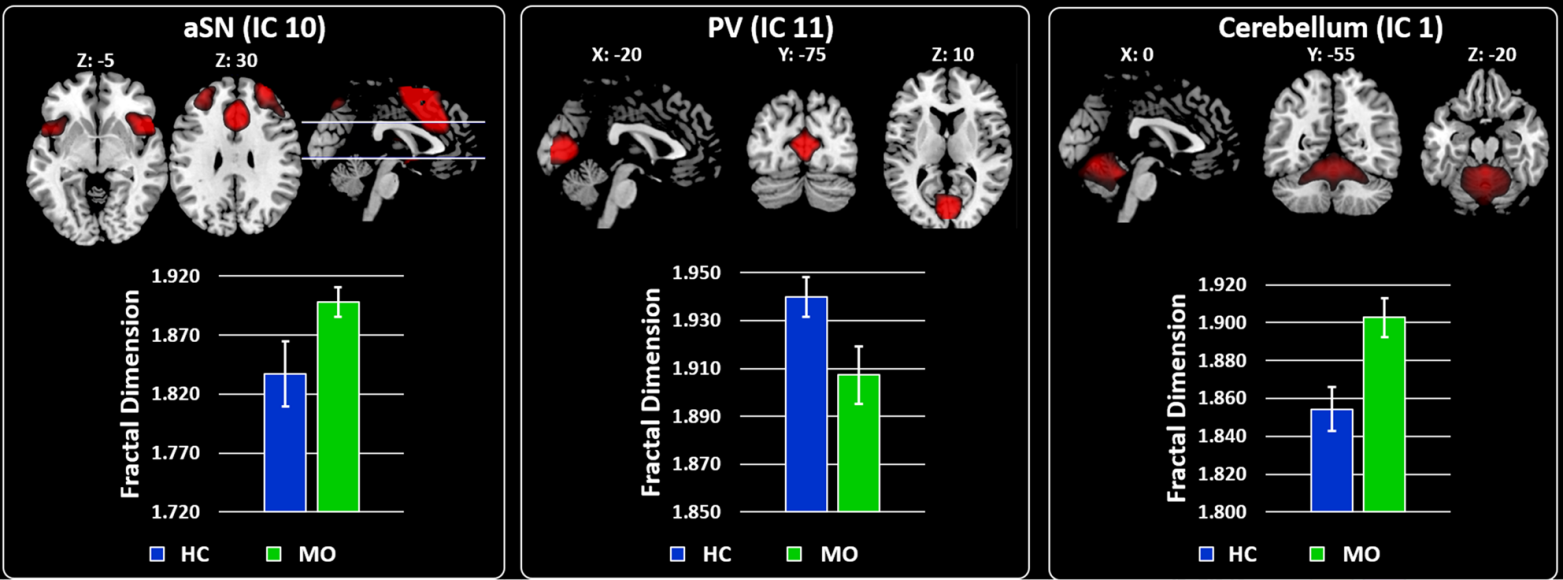
RSNs BOLD characterization by Higuchi’s fractal dimension (FD). For each panel (Left, Middle and Right)—Spatial maps of the IC obtained by GIFT toolbox. Grand average and standard error for the FD values (*k* = 12) are shown for both groups HC (blue) and MO (green). Left panel—Shows the results obtained for IC10 representing the aSN. Middle panel—Shows the results for IC 11 representing the PV. Right panel—Shows the results for IC 1 representing the Cerebellum. All images have been co-registered into the Montreal Neurological Institute (MNI) space. The numbers above each image refers to the X, Y and Z coordinates in MNI space. *aSN* anterior part of the salience network, *PV* primary visual network.

The rm-ANOVA model for FD values revealed that the interaction effect *GROUPs* × *ICs* was significant (Wilks’ *λ* = 0.262, F_19,20_ = 2.962, p = 0.010). Because the sphericity assumption was violated (Mauchly’s W < 0.001, ${\chi }_{189}^{2}$ = 307.650, p < 0.001), the *ε* adjustment was adopted in the univariate test for repeated measures, which resulted significant (F_9,117_ = 1.936, *ε* = 0.480, p = 0.045). At univariate level, MO differed from HC in FD values of IC1/Cb (F_1,38_ = 10.638, p = 0.002), IC10/aSN (F_1,38_ = 4.842, p = 0.034) and IC11/PV (F_1,38_ = 4.716, p = 0.036). Compared to the HC, higher FD values were observed in MO for IC10/aSN (*d* = 0.80; Fig. 2—left panel) and IC1/Cb (*d* = 0.84; Fig. 2—right panel). The opposite pattern was observed for IC11/PV (*d* = − 0.76; Fig. 2—middle panel) with lower FD for the MO compared to the HC.

### Correlation analysis

**Figure 3:**
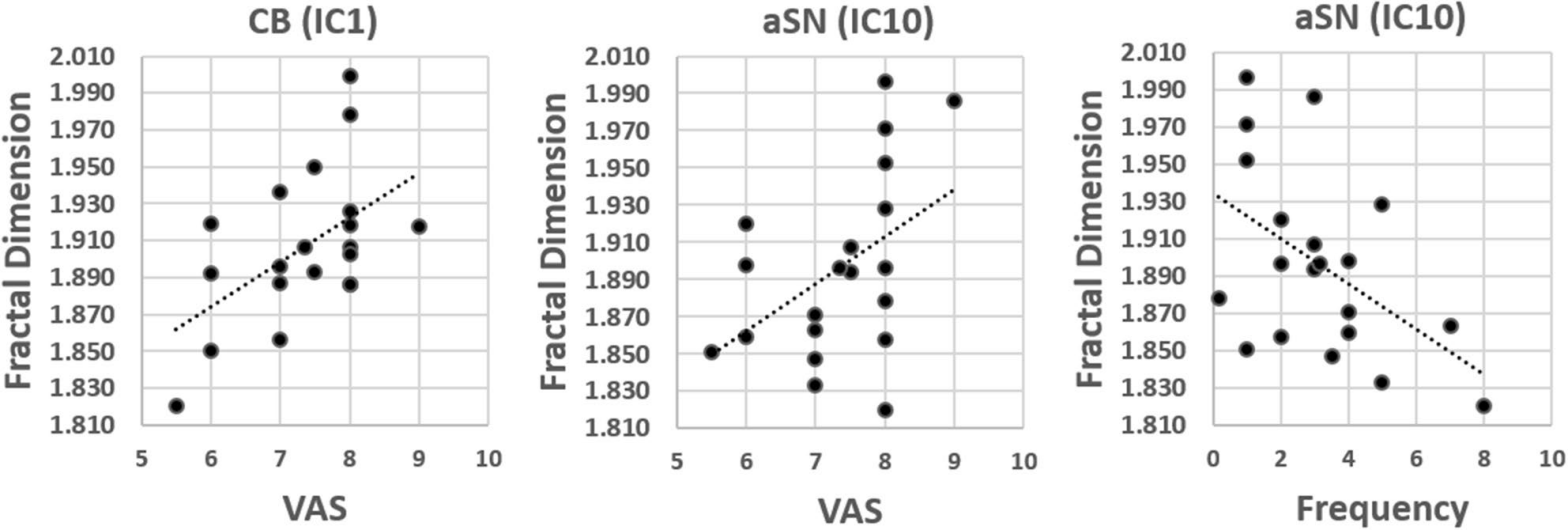
Scatterplots between BOLD IC FD values and clinical variables. Correlation analysis between FD values of IC1 (Cb) and IC10 (aSN) BOLD activity and clinical variables (Frequency and VAS).

Significant correlation was found between severity of migraine pain, as assessed by 0–10 VAS, and FD values of IC1 (*r* = 0.448, p = 0.049) and IC10 (*r* = 0.469, p = 0.037), while attacks frequency correlated with FD values of IC10 (*r* = − 0.486, p = 0.030) (Fig. 3).

No other significant correlation was found between DTI metrics of the hypothalamus, considered as a whole or in its single ROIs, and FD metrics of the networks, as well as with clinical variables.

## Discussion

Confirming our initial hypothesis, we detected an interictal alteration of hypothalamic diffusion-weighted MRI signal in the migraine group. Compared to HC, DTI MRI showed higher values of MD, AD, and RD within the hypothalamus when measured as a single ROI. In migraine patients we also detected significantly higher FD then in the HCs at the level of the salience network and cerebellum, while FD was lower in the primary visual network. Nonetheless, the complexity of cortical networks correlated with attacks frequency and severity of migraine pain. In the exploratory analysis on the contribution of anterior and posterior bilateral hypothalamic ROIs, we detected higher values of MD, AD, and RD within the all ROIs, with lower values of FA in the posterior hypothalamus bilaterally.

### Microstructure of the hypothalamus

The hypothalamus, through its orexinergic and non-orexinergic neuroendocrine system, is a fundamental brain hub devoted to coordinating appropriate physiological and behavioral responses to threatening or potentially threatening internal and external factors^19,20^. For this function, the hypothalamus, particularly in its posterior part, is anatomically connected to the two most important systems of the brain programmed to control pain, the one located in the frontal lobes^21^ and the one located in the midbrain^2,22^. Through these systems, the hypothalamus has an important antinociceptive function^23^.

With the help of modern neuroimaging, some recent studies have supported the historical view^24^ of the hypothalamus as a cerebral structure playing a pivotal role in the recurrence of migraine attacks, its anterior part showing increased neural activity up to 48 h before an attack, in a time period when premonitory symptoms may occur^1–3^. Hypothalamic activation has also been described during the chronic daily or almost daily presentation of migraine attacks, especially in its anterior part when migraine is chronic, and its posterior region during headaches^6, 7, 25^. Volume of the posterior hypothalamic regions in voxel-based morphometry processing was found lower in episodic migraine patients when compared with controls^26^.

Mean, axial, and radial diffusivity were significantly higher in patients than in HC, both considering the hypothalamus as a whole and assessing its anterior and posterior bilateral ROIs separately. The MD metric typically is comprised of RD and AD and quantifies the overall magnitude of water diffusion by indicating both cellular swelling and cellular density^27^. In particular, AD and RD diffusivity is considered to be in vivo surrogate markers of myelin and axonal damage, respectively. We also observed that despite FA DTI metric did not statistically differ between groups when we considered the hypothalamus as a whole, in the exploratory analysis on the hypothalamic ROIs significantly lower values were detected in the posterior parts bilaterally.

The peculiar diffusivity pattern of higher MD, AD, and RD, with lower FA, we found in patients with MO may reflect slightly decreased cellularity (neuronal and glial cells) and/or loss in the directional organization of highly anisotropic myelinated fibers interconnecting individual hypothalamic nuclei in combination with an increased cell density^28^. Indeed, anatomically, the hypothalamus is formed by a conglomerate of grey matter nuclei and by interconnecting myelinated fibers; animal models have indicated that increased cell swelling may coincide with increased in neural electric response^29^.

### Within network fractal dimensionality

The involvement of salience, visual, and cerebellar networks in migraine pathophysiology is not a novel finding. The salience network includes dorsal anterior cingulate and anterior insular cortices^30^. Among its many functions, the SN is involved in self-awareness through the integration of sensory, emotional, and cognitive information^31^. In previous studies performed in MO patients, the SN showed less intrinsic functional connectivity^32,33^ and lack of BOLD response habituation to pain^34,35^ in comparison to HC.

The visual areas probably play a major role in the pathophysiology of migraine. In fact, neurophysiological^36^ and psychophysiological^37,38^ responses, fMRI BOLD evoked activity and spontaneous connectivity^33,39,40^, metabolic activity^41,42^ and structural abnormalities^43^ of the visual areas were all several times found to be altered in patients with episodic migraine when between attacks.

Even though the cerebellum was found to be activated in response to painful stimuli in several studies, it received less attention by researchers who attempt to shed light on migraine pathophysiology^40,44–48^. Its involvement in migraineur pain comes not at odd since it is well known that the deep cerebellar nuclei process noxious stimuli^49–51^ and take part in the perception of pain and its inhibition through their connections with the brainstem and the thalamus^48,52^.

Our findings add a further dimension to the understanding of the pathophysiology of MO; increased fractal dimensionality within the salience and cerebellar networks and decreased FD within the primary visual network could be explained postulating that loss or gain in complexity in brain activity reflects more flexible and/or efficient information processing, as a result of short and long-range interactions between neuronal structures operating at multiple dimensional levels such as space and time^53^. Therefore, the complex pattern found in SN and cerebellum may reflect an increased efficiency demand of brain networks devoted to the integration of sensory, emotional, and cognitive information related to the severity of migraine presentation. In support of this interpretation, we detected that both SN and cerebellum FD correlated positively with the frequency of the recurrence of migraine attacks and with the subjective perception of ictal pain intensity, a datum already described by others^34,48^. The reduced efficiency in information processing we detected within the PV network may be the morpho-functional counterpart of the neurophysiological finding of an initially slower interictal visual cortical reactivity^54^ as well as of the cortical hypometabolism found with the FDG-PET^42^ in between attacks of episodic migraine.

### Relevance for migraine pathophysiology

In recent years, the attention of researchers has shifted from the brain stem to the hypothalamus as a possible generator of migraine. The neuroimaging results obtained longitudinally over a period of 1 month led researchers to believe that the hypothalamus, especially the anterior part, plays a primary role in the beginning of the premonitory phase of the attack^1–3^. Our study shows that the hypothalamus presents structural abnormalities, both in its anterior and posterior regions, even when it is not in a premonitory phase or during migraine attacks. We speculate that these microstructural abnormalities may reflect rising in hypothalamic excitability, and that could be considered as a neuroanatomical substrate favoring the beginning of the prodromal phase and, therefore, the ignition of an attack. The involvement of cortical networks detected by us in the same group of patients could be another favorable factor on which external modifiable factors could further lower the threshold for the activation of the trigeminovascular system which, together with the hypothalamic-pituitary neuroendocrine system, tries to maintain correct homeostasis of the body, i.e. to prevent brain dysexcitability^55^. In this regard, it is interesting to note that most of the areas forming part of the networks analyzed by us were active, together with the hypothalamus, even during the pre-monitory phase of migraine^1^, and that, in migraine families, a gene module in the visual cortex can determine a complex genetic trait setting peculiar gene–gene interactions, transcriptomic, and gene-networking both at the cortical level and at the level of the neuroendocrine system^56^.

Interestingly, our results show no correlation between microstructural metrics of the hypothalamus and cortical complexity metrics in interictal migraine. This could mean both that the hypothalamus is not crucial in the determinism of cortical dysfunction among migraine attacks or that it may become so only when a migraine attack is about to initiate, i.e. at a time when hypothalamus should coordinate appropriate behavioral responses to threatening or potentially threatening internal and external factors^19,20^. Overall, the mechanistic underpinnings of these complex multilevel changes, from structure to function, are still far from being understood as they fit into the complex puzzle of migraine pathophysiology. In the context of this complexity, we note that the area belonging to the networks in which we found abnormal fractal dimensionalities are indeed similar to brain regions in which abnormal cortical hyperresponsiveness to sensory stimuli was previously detected, and therefore considered another factor favoring the repetition of migraine attacks^57^.

A limitation of our study relies in the nature of migraine syndrome, i.e. its high genotypic and phenotypic variability that implies a less immediate generalizability of the results. A larger cohort of patients might allow a better characterization of phenotypic subgroups of migraine patients. Another possible limitation is the lack of correlation between morpho-functional and psychometric variables, such as pain rumination or anxiety.

Future work must investigate the same diffusivity metrics also in the premonitory phase and during migraine attacks. It remains to be seen whether these interictal microstructural anomalies are permanent or can be normalized by pharmacological and non-pharmacological therapies, commonly used for migraine prophylaxis.

## Materials and methods

### Participants

We prospectively enrolled 20 patients affected by episodic migraine without aura (MO) from the headache consultation of Sapienza University of Rome Polo Pontino in Latina (Italy). Patients were initially diagnosed according to the International Classification of Headache Disorders (ICHD) IIIbeta and confirmed according to the ICHD-III^58^. We included only patients who did not have a migraine attack during the scan or in a period between 3 days before or after the scan, as well as those who were not overusing medication or did not have prophylaxis in progress or do it in the 3 months before the scan. We recruited 20 healthy subjects with no personal or family history of migraine or other primary headaches as controls (HC). For all participants in the study, the exclusion criteria were the presence of other concomitant neurological disorders, or obvious psychiatric, endocrinological, autoimmune, connective tissue disorders, and ophthalmological disorders as assessed with a complete neuro-ophthalmological evaluation including best-corrected visual acuity, slit-lamp biomicroscopy, intraocular pressure measurement, and indirect ophthalmoscopy. This study is part of a larger one performed between 2013 and 2019, in which the same participant underwent multiple neuroimaging procedures during the same experimental session. All the scanning sessions took place during the afternoon. Women who participated in the study were scanned outside the days of menstruation (11.4 ± 4.0 days from menstruation onset in HCs, 12.0 ± 3.9 in MO patients). All participants were first informed about the aims of the study and the procedures, then signed informed consent. The study was approved by the ethical committee of the Sapienza University of Rome (RIF.CE 4839). All experiments were performed in accordance with the Declaration of Helsinki.

### fMRI data acquisition and preprocessing

MRI data were obtained on a Siemens 3 T Verio scanner using a 12-channel head coil. Structural anatomic scans were performed using a T1-weighted sagittal magnetization-prepared rapid gradient echo (MPRAGE) series (TR: 1900 ms, TE: 2.93 ms, 176 sagittal slices, 0.508 × 0.508 × 1 mm^3^ voxels)^15^. We acquired an interleaved double-echo Turbo Spin Echo sequence proton density and T2-weighted images (repetition time: 3320 ms, echo time: 10/103 ms, matrix: 384 × 384, field of view: 220 mm, slice thickness: 4 mm, gap: 1.2 mm, 50 axial slices). Diffusion tensor imaging (DTI) was acquired by using single-shot echo-planar imaging, with a 128–channel head coil (TR 12,200 ms, TE 94 ms, 72 axial slices, 2 mm thickness, isotropic voxels). Images from the same participants and during the same session were obtained with diffusion gradients applied along 30 non-collinear directions, effective b values of 0 and 1000 s/mm^2^ were used. Functional MRI data were obtained using T2*-weighted, echo-planar imaging (TR: 3000 ms, TE: 25 ms, 40 axial slices, 3.906 × 3.906 × 3 mm, 150 volumes). Functional resting scans lasted seven minutes and 30 s, during which participants were instructed to relax, avoid motion and keep their eyes closed, but not to fall asleep.

Data pre-processing was carried out using SPM12 software (http://www.fil.ion.ucl.ac.uk/spm/) implemented in MATLAB (version R2016b, MathWorks, Inc., Natick, MA, USA). Data were realigned to the first volume to correct for head motion using a 6-parameter rigid body process and resliced by cubic spline interpolation. The structural (T1-MPRAGE) and functional data were co-registered for each participant dataset. Normalization procedure transformed structural and realigned EPI images into a common stereotactic space based on Talairach and Tournoux^59^, resampled by 3 mm in each direction. Finally, the spatially normalized functional images were smoothed isotropically at 8 × 8 × 8 mm.

### Diffusion tensor imaging (DTI) analysis

FSL 6.0 software package (FMRIB Image Analysis Group, Oxford, England; https://fsl.fmrib.ox.ac.uk/fsl/fslwiki) was used to process image data.

The FSL Diffusion Toolbox (FDT) was used to correct susceptibility induced distortions^60^, eddy currents^61^, and motion artifacts^62^, while the brain extraction tool (BET) was used to create brain masks from the b0 image of each participant^63^. An automated quality control framework was used to assess diffusion MRI data^64^.

The FSL toolbox DTIFIT fits the pre-processed image based on a diffusion tensor model to yield AD (axial diffusivity), FA (fractional anisotropy), MD (mean diffusivity), and RD (radial diffusivity).

For each subject, a region of interest (ROI) was defined, which covers the whole of the hypothalamus. In addition, for each subject we defined 4 further regions of interest covering the left and right anterior hypothalamus—mostly with neuroendocrine function—and the left and right posterior hypothalamus, including the wake promoting nuclei. For this purpose, we used the coordinates provided by Boes et al.^65^. The size of the hypothalamic ROI in the 2 mm space was 6 voxels, 3 per hemisphere, equally for the anterior and posterior ROIs. Mean AD, FA, MD, and RD values in the hypothalamus for every subject were calculated by averaging those voxels in the ROI.

### fMRI data analysis

After data preprocessing, resting-state data of all participants were concatenated (HC and MO) and analyzed using spatial independent component analysis (ICA) based on the infomax algorithm, as implemented in the Group ICA of fMRI Toolbox (GIFT—http://trendscenter.org/software/gift/) to decompose the data into functional networks that exhibited a unique time course profile. Two data reduction steps were carried out using principal component analysis, subject-specific and group-level steps. Firstly Subject-specific data were reduced to 50 components and subsequently reduced data were concatenated over time. Secondly, at group level, data were reduced into 36 group independent components (ICs) using the expectation–maximization algorithm included in GIFT^66^.

Spatial ICAs was also performed separately for HC and MO patients to ensure that the resulting components had similar resting-state fluctuations in the two groups as in the resulting components obtained from all 40 participants combined.

The number of ICs was estimated using the minimum description length (MDL) criterion^67,68^. In our specific case, 33 independent components (ICs) were indicated to be estimated. Subject-specific spatial maps and time courses were obtained using the back-reconstruction approach (GICA)^69^.

From the 33 ICs, we identified the relevant RSNs by applying a previously described procedure^66^. Two experienced neuroradiologists (E.T. & F.C.) blindly reviewed the components discarding those showing spatial overlap with vascular, ventricular, edge regions corresponding to artifacts^70^. This process resulted in 20 meaningful ICs that we sorted into 10 functional networks, based on the spatial correlation between independent components and the template provided by GIFT Toolbox^66^. The functional networks were arranged into (Fig. 1): Cerebellum (Cb—IC1); Auditory Network (AN—IC2); Fronto-Parietal Network (FPN—IC3 and IC23); Dorsal Attention System (DAS—IC6 and IC17); Sensory Motor Network (SMN—IC7, IC8, IC33); Salience Network (SN—IC 10 and IC19); Visual Network (VN—IC11 and IC 24); Default Mode Network (DMN—IC15, IC20, IC28, and IC31); Precuneous (IC25 and IC27) and Language Network (LN—IC 26).

### Characterization of the BOLD RSNs by Higuchi’s fractal dimension

Higuchi’s fractal dimension (FD)^11^ is a nonlinear measure of waveform complexity in the time domain. Discretized functions or signals could be analyzed as time sequences X(1), X(2), …, X(N), where N is the total number of samples. From the starting time sequence, a new self-similar time series X_m^k can be calculated as:

$${X}_{m}^{k}:x\left(m\right), x\left(m+k\right), x\left(m+2k\right),\dots ,x(m+int\left(\frac{N-k}{k}\right)k)$$

for m = 1, 2, …, k where m is the initial time; k is the time interval, k = 1, 2, …, kmax; kmax is a free parameter, and int(r) is the integer part of the number r.

The length, Lm(k), of each curve Xkm is calculated as:

$$L_{m} (k) = \frac{1}{k}\left[ {\sum\limits_{{i = 1,{\text{int}} \left( {\frac{N - m}{k}} \right)}} {\left| {X(m + ik) - X(m + (i - 1)k)} \right| \cdot \frac{N - 1}{{{\text{int}} \left( {\frac{N - m}{k}} \right)}}} } \right]$$

where N is the length of the original time series X and (N − 1)/{int[(N − m)/k]k} is a normalization factor. Lm(k) was averaged for all m forming the mean value of the curve length L(k) for each k = 1, …, kmax as:

$$L\left(k\right)=\frac{\sum_{m=1}^{k}{L}_{m}(k)}{k}$$

An array of mean values L(k) was obtained, and the FD was estimated as follow:

$${\text{FD}} = {\text{ln}}\left( {{\text{L}}\left( {\text{k}} \right)} \right)/{\text{ln}}\left( {{1}/{\text{k}}} \right) \, \;\;\;\;\;\;{\text{for k}} = {1},{ 2}, \, \ldots ,{\text{ k}}_{{{\text{max}}}}$$

In practice, the original curve or signal can be divided into smaller parts with or without overlap, called “windows”. Then, the method for computing FD should be applied to each window where N should be seen as the length of the window. In that case, FD values are calculated for each window, with or without overlap. Individual FD values can be averaged across all windows for the entire curve, and the mean FD value can be used as a measure of curve complexity.

Here, using the single-subject IC time courses for each RSN, we calculated FD in non-overlapped time windows of 150 s (corresponding to 50 of our fMRI volumes). The choice of the free parameter k has a crucial role in FD estimation. For each window we estimated twenty-four values of FD for k = 2, …, 25. The value 25 was equal to half of the samples within our 50 volumes window (i.e. 150 s). kmax is equal to half of the window length the maximum length that can be chosen. There were three windows within our 150 volume scans, therefore we estimated three measures of FD at each value of k (e.g. FD2, FD3, FD4, …., FD24). These three measures were averaged to give one mean value of FD for each k, for each subject^12,17,71^. The process was then repeated for every subject and every RSN. Higuchi's (FD) can be seen as a quantitative nonlinear measure of the BOLD signal dynamics^15,16^.

### Sample size calculation

As our primary endpoint was to detect differences in the hypothalamic microstructure between HC and MO, a sample size calculation was based on pilot data from 20 subjects, ten for each group, enrolled independently from the current study. For HC, FA was 0.282 ± 0.049, MD 1.134E−03 ± 1.704E−04, AD 1.404E−03 ± 1.707E−04, and RD 9.944E−04 ± 1.772E−04. For MO, FA was 0.232 ± 0.032, MD 1.371E−03 ± 1.221E−04, AD 1.647E−03 ± 1.164E−04, and RD 1.237E−03 ± 1.251E−04. Assuming that the values in each subject group were normally distributed with a within-group SD of 0.048 for FA, 1.887E−04 for MD, 1.891E−04 for AD, and 1.943E−04 for RD, to fulfill the desired power of 90% with the significance level at 5%, the required sample size was for FA 20 subjects for each group, for MD 14, for AD 14, and for RD 15. To be more conservative, we decided to complete the enrollment when 20 subjects for each group were scanned in MRI.

### Statistical analysis

Kolmogorov–Smirnov test for normality indicated that DTI metrics of hypothalamus and its 4 regions of interest and FD values of all the twenty retained ICs did not differ from a Gaussian distribution (consistently, p > 0.200).

In order to control the type I error rate due to the multiple comparisons, we carried out a model of multivariate analysis of variance (MANOVA), a model of repeated-measures of multivariate analysis of variance (rm-MANOVA), and a model of repeated-measures analysis of variance (rm-ANOVA) on the DTI metrics of the hypothalamus, the DTI metrics the four regions of interest of hypothalamus, and the FD values, respectively. MANOVA, followed by univariate ANOVAs, was employed to investigate the *GROUPs* effect (between-subject factor: *HC vs. MO*) on AD, FA, MD, and RD (dependent variables). As the presence of outliers can increase type I error rate in MANOVA, Mahalanobis Distance was used to identify potential multivariate outliers. Mahalanobis Distance critical value of chi-square distribution, for degrees of freedom = 4 and p < 0.001, was 18.47. Univariate ANOVA results were examined only if Wilks' Lambda multivariate significance criterion was satisfied. rm-MANOVA, followed by univariate rm-ANOVAs, was employed to investigate the *PARTs* × *SIDEs* × *GROUPs* interaction effect (*PARTs* and *SIDEs* are the two within-subject factors: respectively, *anterior vs*. *posterior* part of hypothalamus and *left vs*. *right* hypothalamus; *GROUPs* is the between-subject factor: *HC vs. MO*) on FA, MD, AD, and RD (dependent variables). For the rm-MANOVA, Mahalanobis Distance critical value of chi-square distribution, for degrees of freedom = 16 and p < 0.001, was 39.25. As for MANOVA, univariate ANOVA results were analyzed only if the Wilks’ Lambda multivariate significance criterion was achieved. rm-ANOVA was performed on the FD values to investigate the interaction effect *GROUPs* × *ICs* (the two *GROUPs* as a between-subject factor: *HC vs. MO*; the twenty *ICs* as a within-subjects factor: *IC1 vs. IC2 vs. IC3 vs. IC6 vs. IC7 vs. IC8 vs. IC10 vs. IC11 vs. IC15 vs. IC17 vs. IC19 vs. IC20 vs. IC23 vs. IC24 vs. IC25 vs. IC26 vs. IC27 vs. IC28 vs. IC31 vs. IC33*) (Fig. 1). As for MANOVA and rm-MANOVA, univariate ANOVA results were analyzed only if the Wilks’ Lambda multivariate significance criterion was achieved. The sphericity of the covariance matrix was verified with the Mauchly sphericity test. In the case of violation of the sphericity assumption, the Greenhouse–Geisser epsilon adjustment was used. Cohen’s *d* was used as a measure of effect size in univariate ANOVAs of MANOVA, rm-MANOVA, and rm-ANOVA models.

Analysis of Pearson correlation coefficient was performed respectively between FD values for each IC and DTI metrics values for hypothalamic ROIs and clinical variables (e.g.: the severity of headache attacks, ranging 0 to 10; the duration of migraine history, in years; the number of monthly migraine attacks; the number of days from the last migraine attack; the monthly number of acute medications).

The significance threshold was set at a p-value < 0.05.

## Data Availability

Clinical, imaging and statistical data will be available upon request from any qualified investigator.
